# Hypomagnetic Field Exposure Alters Iron–Sulfur Homeostasis and Oxidative Balance in a Frataxin-Deficient Insect System

**DOI:** 10.3390/insects17040373

**Published:** 2026-04-01

**Authors:** Hui-Ming Kang, Bing Li, Shuai Yan, Li-Li Zhang, Gui-Jun Wan, Jun-Zheng Zhang, Wei-Dong Pan

**Affiliations:** 1 Beijing Key Laboratory of Bioelectromagnetics, Institute of Electrical Engineering, Chinese Academy of Sciences, Beijing 100190, China; kanghuiming@mail.iee.ac.cn (H.-M.K.); libing@mail.iee.ac.cn (B.L.); 2 School of Electronic, Electrical and Communication Engineering, University of Chinese Academy of Sciences, Beijing 101408, China; 3 Shanghai Synchrotron Radiation Facility, Shanghai Advanced Research Institute, Chinese Academy of Sciences, Shanghai 201204, China; yanshuai@sari.ac.cn (S.Y.); zhanglili@sari.ac.cn (L.-L.Z.); 4 Department of Entomology, College of Plant Protection, Nanjing Agricultural University, Nanjing 210095, China; guijunwan@njau.edu.cn; 5 Department of Plant Biosecurity and MOA Key Laboratory of Surveillance and Management for Plant Quarantine Pests, College of Plant Protection, China Agricultural University, Beijing 100193, China; zhangjz@cau.edu.cn

**Keywords:** hypomagnetic field, frataxin, SR-XRF, iron/sulfur, oxidative stress

## Abstract

Frataxin is a conserved mitochondrial protein essential for cellular iron–sulfur (Fe–S) cluster biogenesis and oxidative balance, with its deficiency causing Friedreich’s ataxia in humans. The hypomagnetic field (HMF), an environmental stressor known to influence oxidative stress and neurodevelopment, may interact with such inherent metabolic vulnerabilities. This study investigated whether HMF exposure exacerbates Fe–S homeostasis and oxidative disruption in a *Drosophila melanogaster* model of frataxin deficiency. Using synchrotron radiation-based X-ray fluorescence (SR-XRF) spectroscopy for in situ elemental analysis in live tissues, we found that HMF significantly altered iron distribution and content in a tissue-specific manner. In frataxin-silenced brains, HMF decreased iron distribution but increased total iron content, whereas in eyes it reduced iron content. Sulfur content decreased in frataxin-deficient eyes but increased in brains under HMF, though its spatial distribution was unchanged. Critically, HMF elevated reactive oxygen species (ROS) in frataxin-deficient brains. Transcriptomic analysis identified 202 differentially expressed genes under HMF in frataxin-silenced flies, including key regulators of iron metabolism and oxidative stress pathways. These findings demonstrate that HMF disrupts tissue-specific iron and sulfur homeostasis and intensifies oxidative stress in a frataxin-deficient insect system, underscoring its role as an environmental factor capable of aggravating metabolic fragility.

## Simple Summary

This study explored whether a hypomagnetic field, an environmental condition found in some places like deep space or shielded rooms, could worsen the problems associated with a genetic disease called Friedreich’s ataxia. This disease is caused by a lack of the vital protein frataxin, which is essential for cellular iron–sulfur cluster biogenesis and oxidative balance. Using fruit flies as a model, researchers aimed to see if this hypomagnetic field would further disturb these elements and increase stress. They found that the hypomagnetic field changed iron and sulfur levels in very specific ways in different tissues, like the brain and eyes, and significantly increased harmful molecules in the brains of affected flies. It also altered the activity of genes related to iron and stress. The conclusion is that this environmental factor can indeed intensify the core cellular damage in this condition. This is valuable to society because it highlights how certain environmental conditions could potentially worsen symptoms for patients with similar metabolic diseases, informing future protective strategies.

## 1. Introduction

Frataxin, a nuclear-encoded mitochondrial protein, is universally recognized as a critical factor in iron–sulfur cluster (ISC) biogenesis and cellular iron homeostasis [1,2,3,4,5,6]. Its deficiency is the primary cause of Friedreich’s ataxia (FRDA), a common hereditary neurodegenerative disease. Although its precise molecular functions are still being elucidated, a leading hypothesis posits that frataxin acts as an iron chaperone, serving as a finely tuned “gatekeeper” that delivers ferrous iron to the scaffold protein ISCU and prevents premature or ineffective reactions during ISC assembly. Iron–sulfur clusters are vital cofactors for numerous metalloproteins involved in electron transport, enzymatic catalysis, and gene regulation. The consequences of frataxin deficiency are profound and conserved across model organisms, leading to mitochondrial iron accumulation, cytoplasmic iron depletion, reduced activity of Fe-S-dependent enzymes, and increased oxidative stress [7,8]. This cascade of mitochondrial dysfunction manifests clinically as impaired motor coordination, reduced lifespan, and, in the case of *Drosophila melanogaster* (*D. melanogaster*), a specific developmental blockade [9,10].

The pathological cascade involved in frataxin deficiency is complex, culminating in late-stage mitochondrial iron accumulation. In *D. melanogaster* models, this iron directly drives neurodegeneration by activating the sphingolipid metabolism-Pdk1/Mef2 signaling axis—a pathway also conserved in mammalian hearts [11]. Given frataxin’s established role in mitochondrial iron handling and redox metabolism, it is of interest to examine how frataxin deficiency responds to environmental perturbations that may affect these same processes. One such perturbation is the hypomagnetic field (HMF), a condition of markedly reduced magnetic flux encountered in deep-space and magnetically shielded environments. Rather than representing a direct patient-relevant exposure scenario, HMF is used here as an experimental environmental stressor to probe whether reduced magnetic field conditions can modify redox balance, mitochondrial-associated function, and iron-related homeostasis in a metabolically vulnerable background. In this context, *D. melanogaster* provides a tractable model for testing how environmental conditions interact with frataxin deficiency at the tissue level.

We therefore used synchrotron radiation-based X-ray fluorescence (SR-XRF) spectroscopy to characterize iron- and sulfur-associated signals in living tissues of *D. melanogaster* under GMF and HMF conditions. In parallel, we assessed oxidative-stress-associated fluorescence and transcriptomic responses to determine whether HMF exposure modifies molecular and cellular readouts in a frataxin-deficient background. Our aim was to evaluate HMF as an environmental perturbation model for studying gene–environment interactions relevant to metabolic fragility, rather than to imply a direct clinical exposure model for Friedreich’s ataxia. Given that iron dyschondrosteosis is a pathological hallmark of FRDA [12]—initially revealed by granular iron deposits in cardiomyocytes—understanding the subcellular spatial distribution of iron is critically important. To this end, we are leveraging the unique capabilities of synchrotron radiation-based X-ray fluorescence (SR-XRF) spectroscopy. The SR-XRF method is ideally suited for acquiring quantitative, spatially resolved data on spatially resolved elemental signals with high resolution [13]. This study aims to use these insights to investigate the spatiotemporal dynamics of iron metabolism under the environmental stress of a hypomagnetic field, using *D. melanogaster* as a model organism. We will employ microbeam XRF and perform real-time, quantitative analysis of iron and sulfur content, distribution, and chemical states in live tissues of *D. melanogaster*. Furthermore, in order to explore the differential gene expression under hypomagnetic field exposure in frataxin-deficient flies, the comparative transcriptomes of frataxin-silenced and parental lines were sequenced using RNA-sequencing (RNA-Seq) technology and comparative analyses were performed to uncover the targeted alterations in genetic networks governing cellular oxidative stress responses and iron ion metabolism. Our goal was to elucidate the precise function and molecular pathways of frataxin in mediating these changes, thereby advancing our understanding of FRDA pathogenesis and opening avenues for new therapeutic interventions.

## 2. Materials and Methods

### 2.1. Drosophila Melanogaster Lines and Cultivation

The Gal4-UAS system was utilized for tissue-specific gene expression. The tub-Gal80^ts^/CyO; repo-GAL4/TM6B lines obtained from China Agricultural University were used to drive RNAi expression. The RNAi line used in this work was UAS-*fh* RNAi (Bloomington Line Center, BDSC 24620, Bloomington, IN, USA). *D. melanogaster* stocks were cultivated at 25 ± 1 °C, with a relative humidity of 60 ± 5% and a 12:12 h light: dark cycle. All magnetic field exposure assays were performed at 25 °C. For genotypes carrying tub-GAL80^ts^ (repo-GAL4; tub-GAL80^ts^ > *fh*-RNAi), flies were reared at 18 °C during development and shifted to 29 °C at 3–5 days post-eclosion to induce adult-onset RNAi, followed by magnetic field exposure at 25 °C. In contrast, the ninaE-GAL4 > *fh*-RNAi flies (without tub-GAL80^ts^) were maintained at 25 °C throughout the whole experiment. Standard regular diet (RD): agar 20 g/L, sucrose 80 g/L, active dry yeast 5 g/L, calcium chloride dihydrate 1.6 g/L, ferrous sulfate heptahydrate 1.6 g/L, sodium potassium tartrate tetrahydrate 8 g/L, sodium chloride 0.5 g/L, manganese chloride tetrahydrate 0.5 g/L, and nipagin 5.3 mL/L [14].

### 2.2. Construction of Frataxin-Silenced Drosophila melanogaster Mutants Using RNAi

**Figure 1:**
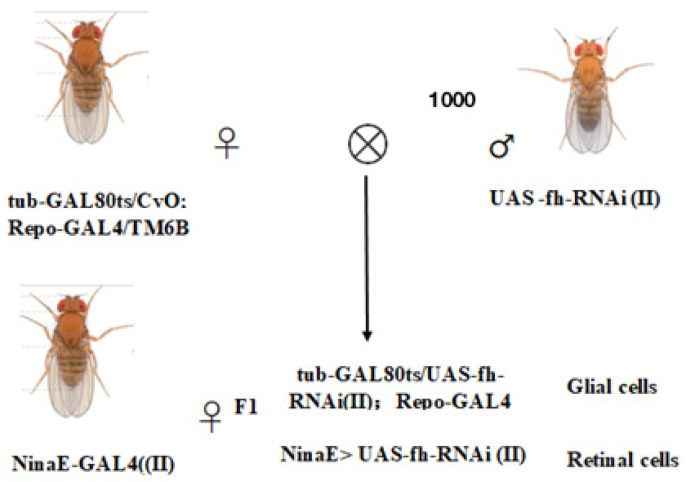
*D. melanogaster* mutants with frataxin silencing in glial and retinal cells.

The GAL4/UAS system was employed to generate frataxin gene silencing in different tissues. The *D. melanogaster* lines were maintained on a cornmeal-agar medium at 25 °C. To achieve time-controlled silencing of frataxin (*fh*) in glial cells (repo-GAL4; tub-GAL80^ts^ > *fh*-RNAi), we employed the GAL4/UAS system combined with tub-GAL80^ts^. In addition, to silence *fh* specifically in retinal photoreceptors, we used ninaE-GAL4 > *fh*-RNAi (without GAL80^ts^) as a retina-specific (non–time-controlled) knockdown strategy. The genetic control was one of the parental lines, UAS-*fh* RNAi. Virgin female flies from one of the parental lines were selected for hybridization. Hybridization experiments were conducted to generate F1 generations with silenced frataxin in glial cells (repo-GAL4; tub-GAL80^ts^ > *fh*-RNAi) and retinal cells (ninaE-GAL4 > *fh*-RNAi) (Figure 1). For all crosses involving GAL80^ts^, both parents and their developmental-stage offspring were reared at 18 °C to suppress GAL4 activity. To induce target gene silencing, F1 offspring were transferred to 29 °C post-eclosion. Upon reaching adulthood, F1 individuals were sorted for specific phenotypes to establish the silencing model.

### 2.3. Magnetic Field Generation System and Insect Exposures

**Figure 2:**
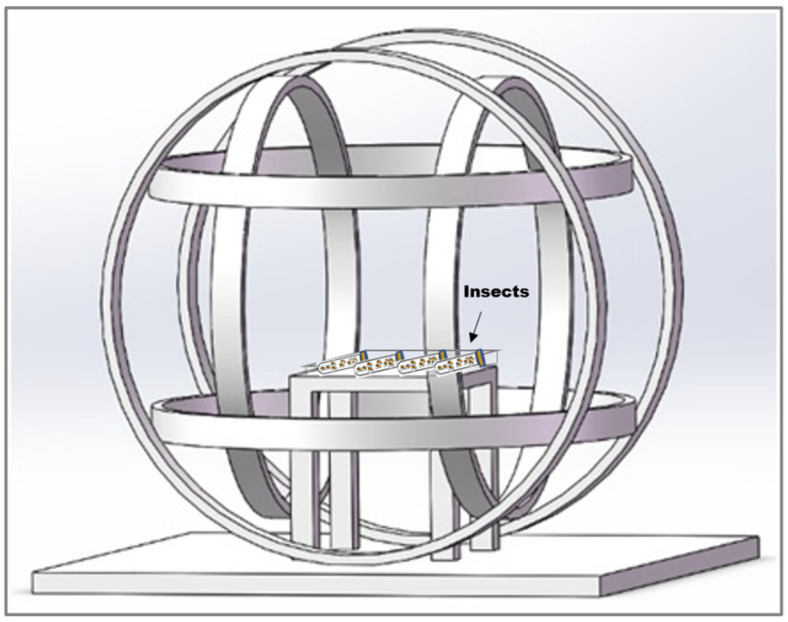
The Helmholtz coil system used to generate hypomagnetic fields (HMF). The system consists of three independent coil pairs, and each pair of coils is individually powered. The external diameter of the apparatus is 1200 mm. The effective area of HMF is generated in the center space, with dimensions of 300 × 300 × 300 mm^3^. Insects are placed on the wooden table in the middle of the Helmholtz coils.

The experiment was conducted in a laboratory in Beijing (39°59′14″ N, 116°19′21″ E), where the local geomagnetic field (GMF) has an intensity of 52,487 ± 841 nT, a declination of 5.30 ± 0.59°, and an inclination of 56°29′ ± 1.02°. To create a hypomagnetic field (HMF), we employed three sets of Helmholtz coils, each powered independently to generate an artificial field that precisely opposed and offset each vector component of the local GMF to nullify the total intensity of the GMF. This setup produced a spherical hypomagnetic environment of 300 × 300 × 300 mm^3^ with an average residual intensity < 500 nT (Figure 2). The external diameter of the HMF system was 1200 mm, and insects were placed on the wooden table in the middle of the Helmholtz coils during experiments. A fluxgate magnetometer (Honor Top model 191A, Qingdao Zhongyu Huantai Magnetoelectric Technology Co., Ltd., Qingdao, China, sensitivity ±1 nT) was used to calibrate and verify the HMF intensity twice daily, both before and after experimental sessions.

*D. melanogaster* flies used for the HMF exposure were 3–10-day-old adult male flies, from *fh*-RNAi flies. The flies were housed in groups of 20 per vial, with three replicate groups per condition. Each condition consisted of three independent biological replicate vials (20 flies per vial) within one experimental cycle. Each experimental cycle consisted of a 72 h exposure to either the HMF or the normal GMF, under a light–dark regimen of 12 h of light followed by 12 h of darkness.

### 2.4. Synchrotron Radiation-Based X-Ray Fluorescence (SR-XRF) and Elemental Distribution and Quantitative Measurements of Iron/Sulfur in Glial and Retinal Cells

To investigate the composition and spatial distribution of iron and sulfur within the microregions of living tissues, brain and eye tissues from *D. melanogaster* male flies exposed to either HMF or GMF conditions were analyzed using microbeam X-ray fluorescence (μ-XRF). The experiments were conducted at the wiggler beamlines 6-2, 10-2 at BL15U1 of the Shanghai Synchrotron Radiation Facility (SSRF) (Shanghai, China). The BL15U1 beamline enables multi-element mapping and quantitative analysis at the sub-parts per million level, utilizing a micron or sub-micron beam. During analysis, the sample was oriented at a 45° angle to the incident X-ray beam, while the X-ray Vortex-90EX Silicon drift detector (SDD) is perpendicular to the beam incidence. The ion chamber is used to monitor the incident photon intensity. An integrated optical microscope facilitated sample observation. The µ-XRF scanning was performed in stepwise mode, with the spectrum for each pixel saved for subsequent quantitative analysis. For data analysis, the software packages GeoPIXE and PyMCA 5.9.7rc1 were employed [15,16,17]. Regions of interest (ROIs) on the spectra can be selected. Once ROIs are created, they are applied across all collected data points to generate two-dimensional, multi-element maps visualizing the distribution of the target elements.

### 2.5. ROS Detection Using DHE Staining and Confocal Microscopy

A 2 mM solution of dihydroethidium (DHE) was prepared using DMSO as the solvent. PBS buffer (500 μL) was combined with 2 μL of DHE stock (2 mM) to achieve a final working concentration of 8 μM. *D. melanogaster* male flies of appropriate genotype and age were selected and transferred using tweezers to a glass 9-well plate containing PBS for dissection under a stereomicroscope. The dissected brain and eye tissues were then placed into a 96-well plate containing DHE solution. The DHE solution was removed, and the samples were washed twice with fresh PBS buffer. Subsequently, 200 µL of 4% PFA solution was added for fixation at room temperature for 5 min. Following suctioning of the fixative, the samples were washed twice with PBS before secondary dissection, and film sealing and imaging were performed. Five biological replicates were conducted for each treatment.

At least 5–10 male flies per genotype were scanned. The samples were analyzed using an LSM 510 confocal microscope (Zeiss, Oberkochen, Germany). All images were acquired using the same exposure time, light intensity, and filter settings across all conditions. Image analysis was performed in Fiji 2.0.0 [18]. For each sample, the region of interest (ROI) was defined consistently for the corresponding brain or eye tissue, and mean fluorescence intensity was measured within the ROI after applying the same analysis workflow across groups. Background signal was measured from an adjacent non-tissue region and subtracted before quantification. Image contrast was adjusted only for visualization and was not used for quantitative measurement.

### 2.6. RNA-Seq and Differential Gene Expression Analysis

Total RNA was extracted from heads of 3–8-day-old adult male flies. For each genotype, three biological replicates with ten heads of flies for each replicate were sent to Novogene for RNA-Seq analysis using TRNzol Universal Reagent (Tiangen Biotech, Beijing, China, Cat. No. DP424) according to the manufacturer’s instructions. RNA quantity and integrity were verified on a Nanodrop 2000 (Thermo, Waltham, MA, USA) for the purpose of library preparation. Sequencing libraries were constructed with the Fast RNA-seq Lib Prep Kit V2 (ABclonal, Wuhan, China, Cat. No. RK20306) following the supplier’s protocol. The clean reads were aligned to the corresponding reference genome using HISAT2 (v2.2.1). Read counts for each gene were obtained with feature Counts (v2.0.6), and gene expression levels were estimated as fragments per kilobase of transcript per million mapped reads (FPKM). Differentially expressed genes (DEGs) between groups were identified using the R Package DESeq2 (v1.42.0), with Benjamini–Hochberg correction to control the false discovery rate. Genes with adjusted *p* value (padj) ≤ 0.05 and |log_2_(FoldChange)| ≥ 1 were considered significantly differentially expressed. Functional enrichment analyses of DEGs were performed using cluster Profiler (v4.8.1) for Gene Ontology (GO) and KEGG pathway enrichment. Gene set enrichment analysis (GSEA) was conducted with the GSEA software (v4.3.2) using default parameters and curated GO/KEGG gene sets.

### 2.7. Statistics

Data analysis was performed using SPSS version 23.0 software (IBM SPSS Statistics Inc., Chicago, IL, USA). A uniform intensity threshold was defined from the background signal of each elemental map, and the area of pixels above this threshold was calculated as a semi-quantitative proxy for the spatial extent of detectable elemental signal. This metric reflects tissue-level signal-positive area and does not resolve subcellular localization, chemical speciation, or definitive intracellular redistribution.

Based on this, normality and homogeneity of variance tests were performed for iron and sulfur content and distribution counts, respectively. When parametric tests were feasible, one-way ANOVA followed by Tukey’s post hoc multiple comparisons was conducted to evaluate the effects of different magnetic fields and genotypes on element content and distribution in brain and eye tissues. The Shapiro–Wilk test was used to test the normality of the data. The data conforming to the normal distribution were expressed as mean ± SEM (standard error of the mean). Outliers were dropped if they were observed due to incorrect entry or measurement of the data. The independent-samples *t*-test was performed to evaluate the effects of different magnetic fields and genotypes on ROS levels in brain and eye tissues. One-way repeated-measures ANOVA with Tukey’s post hoc test was used to measure the effects of frataxin gene silencing on the distribution and content of iron/sulfur in brain or eye tissues of *D. melanogaster* under GMF or HMF. *p* values < 0.05 were considered statistically significant.

## 3. Results

### 3.1. Analysis of Iron/Sulfur Elemental Distribution and Content Based on Frataxin Silencing Model Under GMF or HMF

#### 3.1.1. Iron Elemental Analysis

**Figure 3:**
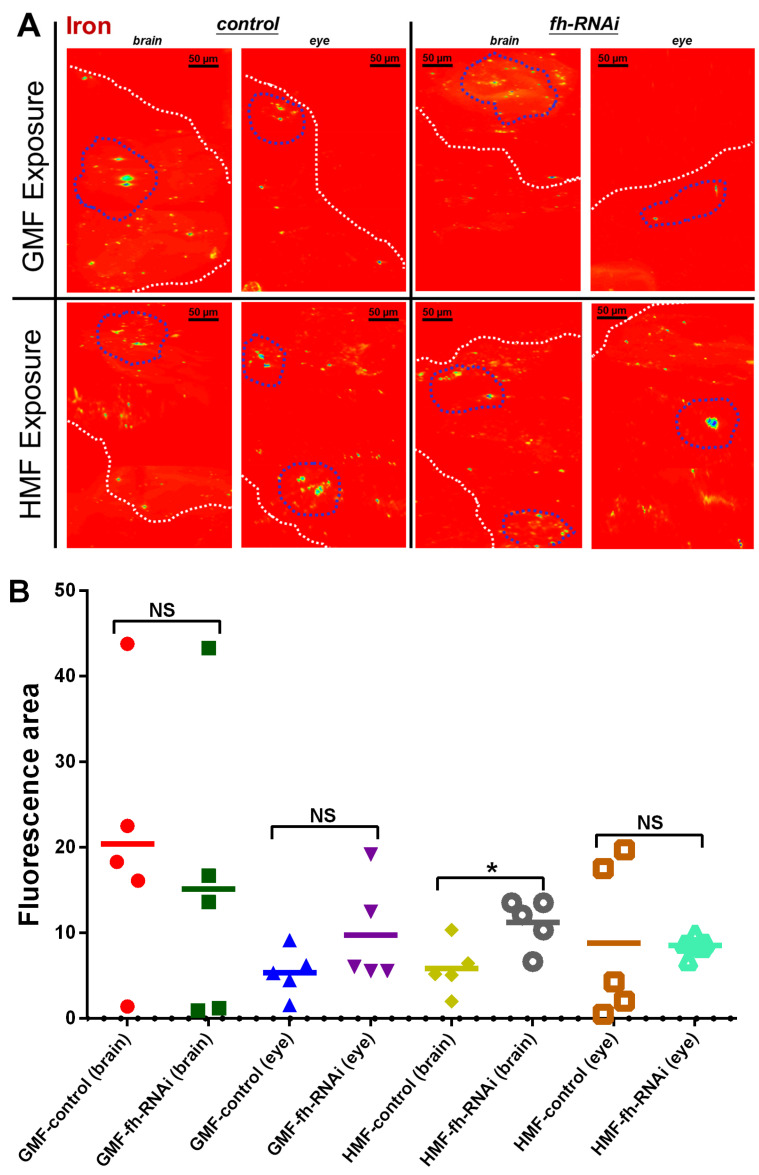
Iron elemental distribution (**A**) and corresponding fluorescence area quantification (**B**) using SR-XRF mapping in brain and eye tissues of *D. melanogaster* under GMF or HMF exposure. The genetic control was UAS-*fh* RNAi, and frataxin silencing in brain and eye tissues were lines of repo-GAL4; tub-GAL80^ts^ > *fh*-RNAi and ninaE-GAL4 > *fh*-RNAi, respectively. Pixel size in the images is 2 μm; scan time is 50 ms. The independent-samples *t*-test (* *p* < 0.05; NS, not significant) was performed to evaluate the effects of different genotypes on fluorescence area in brain and eye tissues under GMF or HMF exposure. Elemental maps were measured in “2D F1Y Scan” mode and normalized to dead time. Note areas with iron hotspots (blue stippled circles) and brain tissue borders (white stippled lines).

**Figure 4:**
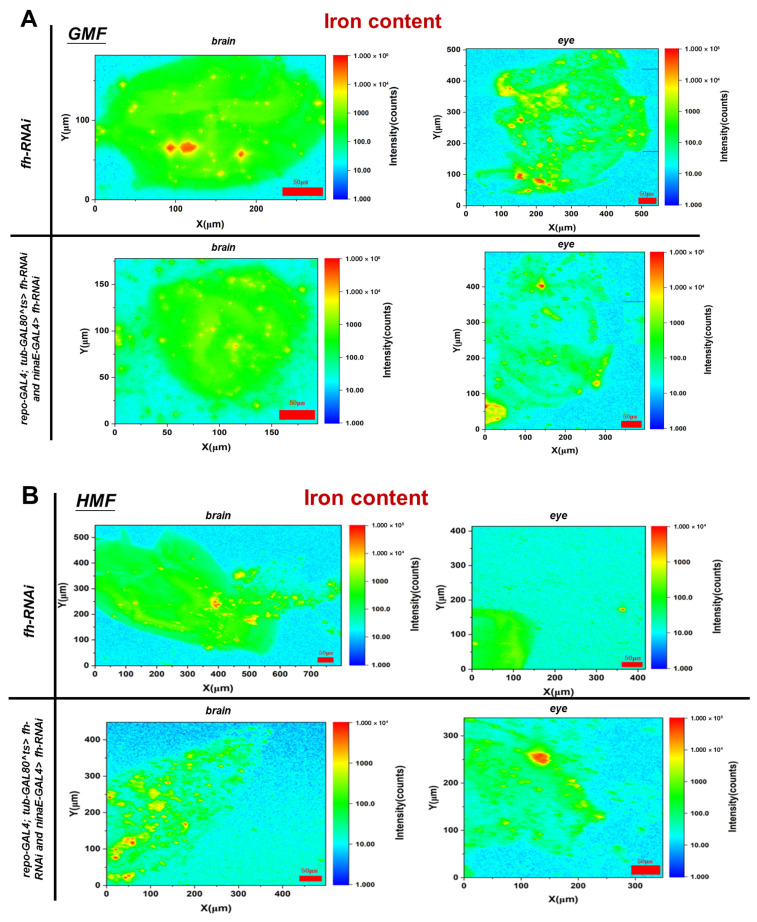
Iron content in brain and eye tissues of *D. melanogaster* after GMF exposure (**A**) or HMF exposure (**B**). The genetic control was UAS-*fh* RNAi, while frataxin silencing in brain and eye tissues was achieved using repo-GAL4; tub-GAL80^ts^ > *fh*-RNAi and ninaE-GAL4 > *fh*-RNAi, respectively. Scale bar: 50 μm. Color code indicates background-corrected intensity of scanned iron in counts.

To determine whether HMF exposure alters iron/sulfur homeostasis in a frataxin-deficient insect system, we examined elemental spatial distribution using SR-XRF mapping (Figure 3A), followed by quantitative assessment of total elemental content (Figure 4). This two-level analysis enables us to unify the spatial distribution and content variations of iron/sulfur elements, thereby obtaining an intuitive understanding of in situ profiles of iron/sulfur elements in the frataxin-deficient model under HMF exposure. In this study, as each brain sample was taken from the whole brain of an insect, the image shows a top-down view of the head from left to right. Compared with the control group, the frataxin-silenced group, either in brain or eye tissue, showed no significant difference in iron elemental distribution (*p* > 0.05) and fluorescence area (*p* > 0.05) under GMF exposure (Figure 3B). However, in brain tissues, compared with the control group, iron elemental distribution was significantly decreased (*p* < 0.05) in frataxin-silenced flies under HMF exposure, and fluorescence area or total fluorescence intensity was significantly increased (*p* < 0.05) in the frataxin-silenced group under HMF exposure (Figure 3B). In contrast, in eye tissues, neither a significant difference in iron elemental distribution (*p* > 0.05) nor in fluorescence area (*p* > 0.05) in frataxin-silenced flies was revealed under HMF exposure (Figure 3B).

Compared with the control group, the iron content in brain and eye tissues after frataxin silencing under GMF exposure decreased by 35.73% (*p* < 0.05) and 16.51% (*p* < 0.05), respectively (Figure 4A). Compared with GMF, the iron content in brain tissues of frataxin-silenced flies increased by 46.71% (*p* < 0.05), while that in eye tissues after frataxin silencing decreased by 24.57% (*p* < 0.05) under HMF exposure (Figure 4A vs. Figure 4B).

#### 3.1.2. Sulfur Elemental Analysis

**Figure 5:**
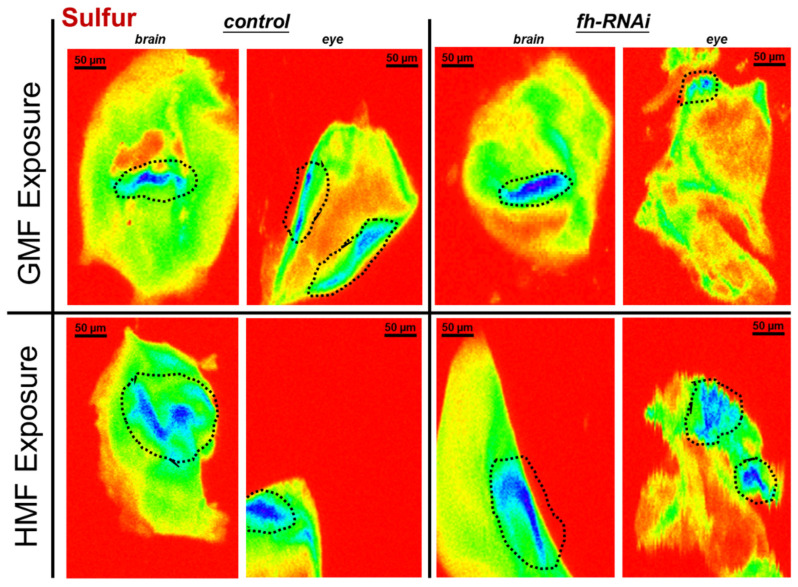
Sulfur elemental distribution in brain and eye tissues of *D. melanogaster* under GMF or HMF exposure. The genetic control was UAS-*fh* RNAi and frataxin silencing in brain and eye tissues were lines of repo-GAL4; tub-GAL80^ts^ > *fh*-RNAi and ninaE-GAL4 > *fh*-RNAi, respectively. Pixel size in the images is 2 μm; scan time is 50 ms. Black striped circles indicate areas with sulfur hotspots (blue ROIs).

Compared with the control group, the frataxin-silenced group showed no significant difference in sulfur signal-positive area (distribution proxy) in brain or eye tissues (*p* > 0.05) and no significant difference in signal-positive area in either tissue (*p* > 0.05). Compared with GMF exposure, frataxin silencing either in brain or eye tissues showed no significant difference in sulfur elemental distribution (*p* > 0.05) or fluorescence area (*p* > 0.05) under HMF exposure (Figure 5).

**Figure 6:**
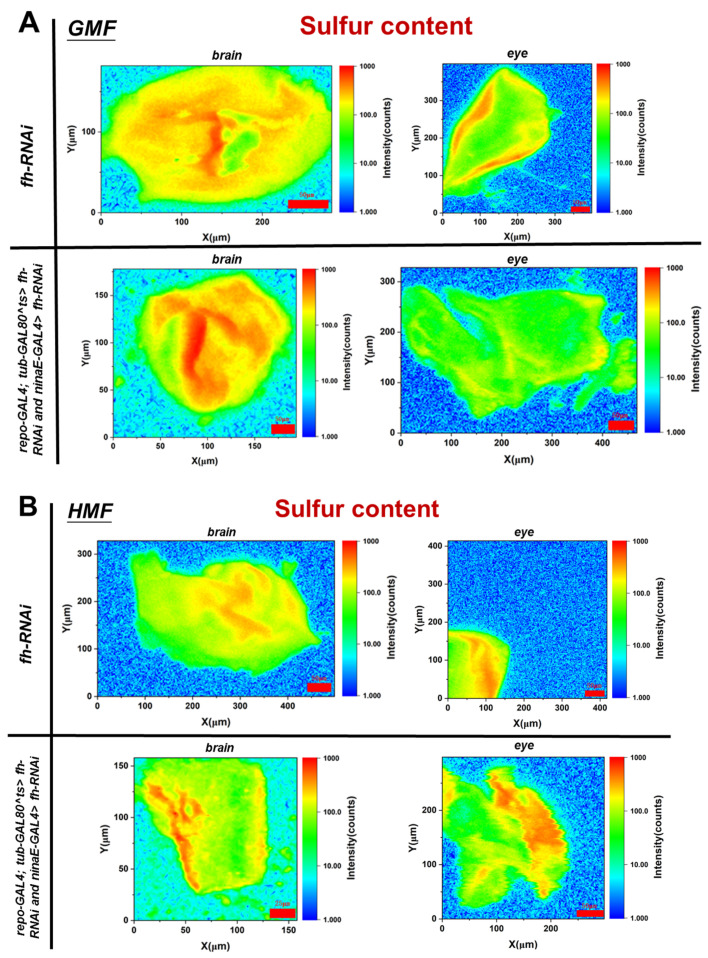
Sulfur content in brain (**A**) and eye (**B**) tissues of *D. melanogaster* under GMF or HMF exposure. The genetic control was UAS-*fh* RNAi and frataxin silencing in brain and eye tissues were lines of repo-GAL4; tub-GAL80^ts^ > *fh*-RNAi and ninaE-GAL4 > *fh*-RNAi, respectively. Scale bar indicates the background-corrected intensity of scanned sulfur in counts.

**Figure 7:**
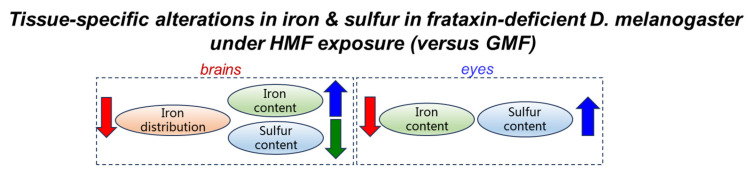
A schematic illustrating tissue-specific alterations in iron and sulfur under GMF vs. HMF conditions. The blue arrow represents the up-regulation, while the red and green arrows represent the down-regulation.

Compared with the control group, the sulfur content in eye tissues after frataxin silencing decreased by 18.76% (*p* < 0.05), and no significant difference in sulfur content in brain tissues was observed with the frataxin-silenced group (*p* > 0.05) (Figure 6A). Compared with GMF exposure, the sulfur content in brain tissues after frataxin silencing was reduced by 40.48% (*p* < 0.05), and that in eye tissues in the frataxin-silenced group was elevated by 65.05% (*p* < 0.05) under HMF exposure (Figure 6B). Together, these data demonstrate that HMF exposure induces tissue-specific alterations in iron/sulfur elemental distribution and total elemental content in a frataxin-deficient insect system (Figure 7), suggesting that magnetic environmental stress may exacerbate iron/sulfur dyshomeostasis through certain mechanisms not yet fully resolved.

### 3.2. ROS Detection Using DHE Staining

DHE is a lipophilic, cell-permeable probe that is itself non-fluorescent. Upon oxidation by intracellular oxidants, it is converted into fluorescent oxidation products. In the present study, DHE fluorescence was used as an indicator of oxidative-stress-associated signal, rather than as a specific readout of any single ROS. Ethidium then binds to DNA, producing red fluorescence (excitation/emission 520/610 nm) that serves as an indicator of intracellular ROS levels. For samples stained with DHE, the mean fluorescence intensity provides a more appropriate measurement of ROS levels.

**Figure 8:**
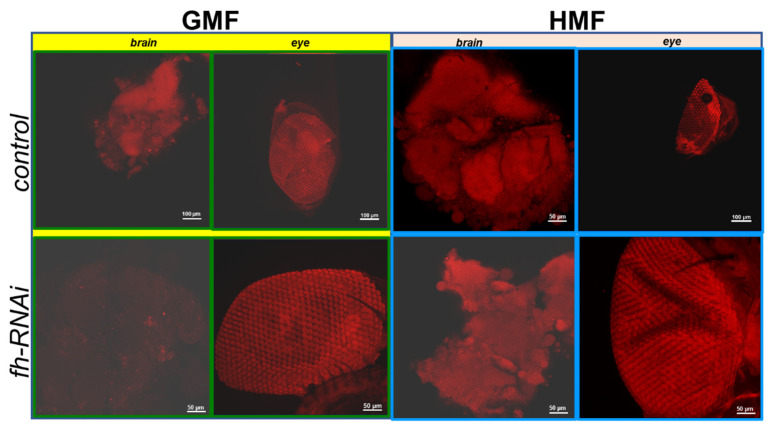
ROS examination of brain and eye tissues of *D. melanogaster* using laser scanning confocal immunofluorescence with dihydroethidium staining. Intracellular ROS levels were visualized as red fluorescence excited from oxidized ethidium. The mean fluorescence intensity serves as an indicator of ROS levels. The genetic control was UAS-*fh* RNAi and frataxin silencing in brain and eye tissues were lines of repo-GAL4; tub-GAL80^ts^ > *fh*-RNAi and ninaE-GAL4 > *fh*-RNAi, respectively.

**Figure 9:**
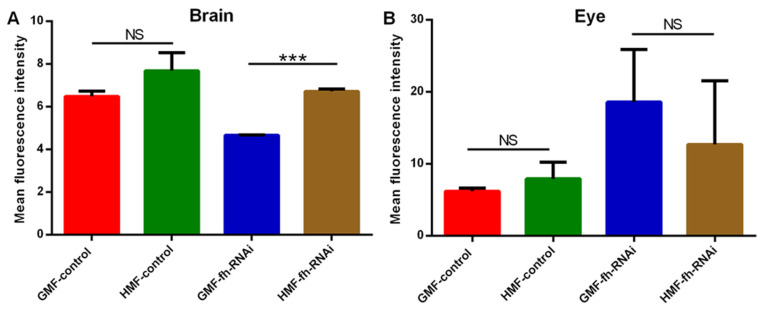
ROS levels of brain (**A**) and eye tissues (**B**) of *D. melanogaster* as indicated by mean fluorescence intensity. The genetic control was UAS-*fh* RNAi and frataxin silencing in brain and eye tissues were lines of repo-GAL4; tub-GAL80^ts^ > *fh*-RNAi and ninaE-GAL4 > *fh*-RNAi, respectively. The independent-samples *t*-test (*** *p* < 0.001; NS, not significant) was performed to evaluate the effects of different magnetic fields or genotypes on ROS levels in brain and eye tissues. Data are presented as the means ± SEM.

As shown in brain tissues (Figure 8 and Figure 9), no significant difference in ROS levels was discovered between the GMF control and the HMF control groups (*p* > 0.05). However, compared with GMF exposure, ROS levels were significantly elevated in frataxin-silenced brain samples under HMF conditions (*p* < 0.001; Figure 9). Corresponding confocal images showed this rise as more intense red fluorescence (Figure 5). In contrast to brain samples, it was found that in eye tissues, compared with GMF exposure, ROS levels revealed no significant difference either with the control group (*p* > 0.05) or with the frataxin-silenced group under HMF exposure (*p* > 0.05) (Figure 9), which was further confirmed by corresponding confocal microscopy (Figure 8).

### 3.3. Analysis of DEGs Under HMF Exposure Specifically in Iron Metabolism, ROS Production and Antioxidation

**Figure 10:**
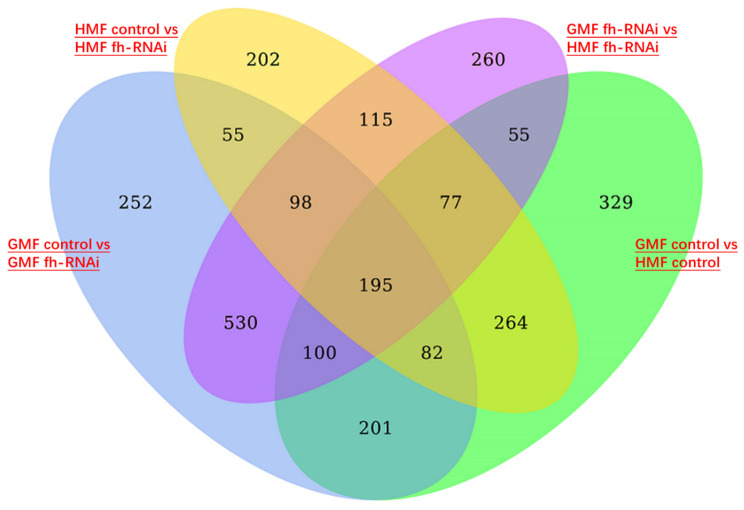
Venn diagram showing four lists of differentially expressed genes in heads of frataxin-silenced flies and in flies exposed to hypomagnetic fields. The genetic control was UAS-*fh* RNAi and frataxin silencing in brain tissues of heads was line of repo-GAL4; tub-GAL80^ts^ > *fh*-RNAi.

A comparison of frataxin-silenced and control flies under HMF exposure identified 202 differentially expressed genes (DEGs), whereas 252 DEGs were identified under GMF exposure (Figure 10). In addition, 260 DEGs were detected between the two frataxin-silenced groups (GMF *fh*-RNAi vs. HMF *fh*-RNAi), and 329 DEGs were detected between the two control groups under different magnetic field conditions (Figure 10). Together, these results indicate that HMF exposure is associated with broad transcriptional shifts in this experimental system. However, these changes may include both primary responses to HMF exposure and secondary downstream stress responses.

Exposure to HMF prompted remodeling of pathways related to iron metabolism and oxidative stress. In the control group, key DEGs involved in iron metabolic regulation, such as cytochrome P450 molecules (6w1/304a1/6d2), were upregulated. ROS sources, including NADPH oxidase and quiescin sulfhydryl oxidase, were also increased, alongside activation of antioxidants like glutathione S-transferase, superoxide dismutase, and peroxidase (Table 1). In contrast, the frataxin-silenced group under HMF showed downregulation of DEGs encoding heme-binding (CG5157) and ferroxidase activities (multicopper oxidase 1/3), as well as reduced ROS-producing enzymes (NADPH oxidase and dual oxidase) (Table 2). Despite the downregulation of peroxiredoxin, antioxidants such as glutathione S-transferase and catalase were upregulated, indicating a transcriptional stress-response pattern. However, these changes should not be interpreted as evidence of functional compensation in the absence of corresponding physiological or biochemical validation (Table 2). To provide a more systematic view of the transcriptomic response, we summarized the major enriched GO and KEGG categories associated with iron metabolism, oxidative stress responses, and related metabolic processes, rather than relying exclusively on selected representative DEGs.

## 4. Discussion

In *D. melanogaster*, frataxin is suggested to maintain mitochondrial Fe-S cluster synthesis and iron metabolic homeostasis, and its deficiency leads to impaired energy metabolism, oxidative stress, and neurodegenerative phenotypes [7,8,19,20,21,22,23]. The discovery of iron deposits in the hearts of FRDA patients in the late seventies [24,25] was the first indication of an association between frataxin and this transition metal, and increased iron content has been reported in critical brain areas of FRDA patients [26,27]. In *D. melanogaster*, Chen et al. showed that iron accumulates in the nervous system in *fh*^1^ mutants [11]. In this study, the role of frataxin in iron metabolism and oxidative stress under environmental stressors such as a hypomagnetic field was investigated primarily using SR-XRF spectroscopy. After frataxin silencing, no significant change either in iron/sulfur elemental distribution or fluorescence area was revealed (Figure 3 and Figure 5). After HMF exposure, however, significant changes happened in iron elemental distribution in frataxin-silenced brain tissues. Moreover, after frataxin silencing, the iron content in both target tissues decreased (Figure 4A), while the iron content in brain and eye tissues of frataxin-silenced flies increased and decreased under HMF exposure, respectively (Figure 4B). The sulfur content showed a similar trend of changes within frataxin silencing (Figure 5), except for the opposite trend of changes in sulfur content in frataxin-silenced tissues after HMF exposure (Figure 6B). Even though total sulfur measurements provide a useful indicator of cellular sulfur status, it should be noted that they do not directly demonstrate Fe–S cluster integrity, as sulfur may exist in various pools including free sulfide, cysteine, and other sulfur-containing metabolites. Nevertheless, these findings suggest that HMF is associated with tissue-specific alterations in iron- and sulfur-associated signals in a frataxin-deficient *D. melanogaster* model. In this context, HMF is best interpreted as an experimental environmental perturbation that modifies elemental readouts in a metabolically vulnerable background, rather than as direct evidence of disease progression in Friedreich’s ataxia.

The knockdown or mutation of *D. melanogaster* frataxin (d*fh*) leads to decreased activity of Fe-S-dependent enzymes (e.g., cis-aconitase, complexes I-III), and it also leads to iron metabolic variations and elevated ROS, especially in neural and muscular tissues [28,29,30]. In *D. melanogaster*, using the *fh*-RNAi line and the actin GAL4-driver line [31,32], the flies with tissue-specific frataxin deficiency in the PNS (C96) or glial cells (repo) showed increased sensitivity to external oxidative stressors such as hyperoxia or H_2_O_2_ treatment [23,31,33]. However, the ROS hypothesis has been questioned in several studies, reporting that loss of FXN leads to hypersensitivity to ROS rather than elevated ROS levels [1,8,28,34,35]. In our study, although a significant change in ROS levels in brain tissues of the control group was not determined under HMF exposure, elevated ROS levels were discovered in the frataxin-silenced group under HMF conditions (Figure 8 and Figure 9). Combined, these data suggest that ROS appears to be more of a tissue-specific “provider of feedback” for HMF rather than a prominent player in model organisms.

A large number of DEGs between frataxin-silenced and control flies under both GMF and HMF conditions further support the strong influence of frataxin deficiency on gene expression related to iron metabolism and oxidative stress. Notably, 260 DEGs were identified between the two frataxin-silenced groups and 329 DEGs between the two control groups under different magnetic field conditions, indicating that HMF exposure is associated with broad transcriptional shifts in this experimental system. These transcriptional changes likely include both primary responses to HMF exposure and secondary downstream stress responses. Although several DEGs related to iron metabolism, ROS production, and antioxidant defense were altered, these findings should be interpreted as evidence of altered transcriptional states rather than proof of functional compensation or a specific protective mechanism.

The proposed methodology of using SR-XRF spectroscopy on live tissues of *D. melanogaster* is a standout feature and improvement over traditional approaches. The ability to perform real-time, quantitative analysis of both iron/sulfur content and distribution at a mutant/parent-type level in a living organism is powerful in this context [17]. This approach promises to move beyond snapshots and provide a “movie” of metabolic alterations, directly testing the gatekeeper hypothesis by observing how the absence of frataxin disrupts the precise choreography of iron and sulfur under stress conditions.

Moreover, we used the pan-glial driver repo-GAL4 instead of the pan-neuronal driver elav-GAL4 to achieve targeted frataxin knockdown because the repo-GAL4 is widely expressed in glial cells, allowing us to simulate frataxin deficiency in nervous system-related tissues while avoiding systemic knockout that could lead to severe developmental defects or fatal outcomes from elav-GAL4–driven neuronal knockdown [36]. As elav-GAL4 drives broad expression early in nervous system development and as *fh* is a core mitochondrial gene, widespread neuronal impairment during development may alter circuit formation, baseline activity, feeding, and developmental rate. In addition, tissue-specific knockdown reduces the risk of developmental mortality that may result from general silencing. Nevertheless, pan-neuronal knockdown could be informative and will be addressed in future work to dissect cell-type contributions.

## 5. Conclusions

In conclusion, our study shows that hypomagnetic field exposure can serve as an environmental perturbation that modifies tissue-specific iron- and sulfur-associated signals, oxidative-stress-associated responses, and gene-expression patterns in a frataxin-deficient insect system. These findings support the use of HMF as an experimental facility for investigating how reduced magnetic field conditions interact with an intrinsically vulnerable metabolic background. Rather than indicating a direct patient-relevant exposure scenario, the present results provide a framework for studying how environmental factors influence redox balance, mitochondrial-associated function, and iron-related homeostasis in the context of frataxin deficiency. By combining a *D. melanogaster* model with live-tissue SR-XRF analysis, this work offers a useful platform for probing gene–environment interactions underlying metabolic fragility.

## Figures and Tables

**Table 1:** Selected differentially expressed genes (DEGs) that are involved in iron metabolism, ROS production and antioxidation in heads of UAS-*fh* RNAi (control) flies after HMF exposure.

Gene_ID	log_2_FoldChange	pval	padj	Gene_Description
FBgn0033065	2.418363708	0	0	Cytochrome P450 6w1(Iron metabolism)
FBgn0038095	4.877657598	9.40 × 10^−200^	1.10 × 10^−197^	Cytochrome P450 304a1 (Iron metabolism)
FBgn0034756	3.355233071	2.80 × 10^−128^	2.05 × 10^−126^	Cytochrome P450 6d2 (Iron metabolism)
FBgn0085428	1.023945	6.22 × 10^−07^	2.93 × 10^−06^	NADPH oxidase (ROS production)
FBgn0038919	1.03	2.40 × 10^−07^	1.19 × 10^−06^	Quiescin sulfhydryl oxidase 2 (ROS production)
FBgn0010041	2.773212	8.10 × 10^−10^	4.92 × 10^−09^	Glutathione S-transferase D5 (Antioxidation)
FBgn0010040	2.7611	5.87 × 10^−05^	0.0002194	Glutathione S-transferase D4 (Antioxidation)
FBgn0051028	1.682223	1.37 × 10^−07^	6.91 × 10^−07^	Related to SOD (Antioxidation)
FBgn0004577	3.186946	4.77 × 10^−05^	0.0001803	Peroxidase (Antioxidation)

Criteria for differentially expressed genes: |log_2_(FoldChange)| > 1 & padj < 0.05.

**Table 2:** Selected differentially expressed genes (DEGs) that are involved in iron metabolism, ROS production and antioxidation in heads of repo-GAL4; tub-GAL80^ts^ > *fh*-RNAi (*fh*-RNAi) flies after HMF exposure.

Gene_ID	log_2_FoldChange	pval	padj	Gene_Description
FBgn0036575	−2.6845	2.45 × 10^−07^	1.18 × 10^−06^	CG5157 (Iron metabolism)
FBgn0032116	−1.89932	1.27 × 10^−104^	6.46 × 10^−103^	Multicopper oxidase 1 (Iron metabolism)
FBgn0039387	−3.35859	1.75 × 10^−07^	8.53 × 10^−07^	Multicopper oxidase 3 (Iron metabolism)
FBgn0085428	−1.92347	3.01 × 10^−18^	2.88 × 10^−17^	NADPH oxidase (ROS production)
FBgn0283531	−1.18372	2.70 × 10^−31^	4.12 × 10^−30^	Dual oxidase (ROS production)
FBgn0033520	−1.3659	0.0003142	0.0010317	Peroxiredoxin 6b (Antioxidation)
FBgn0010041	1.988693	9.08 × 10^−11^	5.71 × 10^−10^	Glutathione S transferase D5 (Antioxidation)
FBgn0010042	2.880674	5.37 × 10^−05^	0.0001985	Glutathione S transferase D6 (Antioxidation)
FBgn0038465	1.271917	5.63 × 10^−307^	1.22 × 10^−304^	Immune-regulated catalase (Antioxidation)

Criteria for differentially expressed genes: |log_2_(FoldChange)| > 1 & padj < 0.05.

## Data Availability

The original contributions presented in this study are included in the article. Further inquiries can be directed to the corresponding author.

## Abbreviations

The following abbreviations are used in this manuscript:

*fh*	Frataxin
SR-XRF	Synchrotron radiation-based X-ray fluorescence
GAL4	GAL gene activator 4
UAS	Upstream activating sequence
GMF	Geomagnetic fields
HMF	Hypomagnetic fields
DHE	Dihydroethidium
DEGs	Differentially expressed genes
